# Biomarkers for Immunotherapy in Poorly Differentiated Sinonasal Tumors

**DOI:** 10.3390/biomedicines10092205

**Published:** 2022-09-06

**Authors:** Eva Villanueva-Fernández, Mario A. Hermsen, Laura Suárez-Fernández, Blanca Vivanco, Alessandro Franchi, Rocío García-Marín, Virginia N. Cabal, Helena Codina-Martínez, Sara Lucila Lorenzo-Guerra, José L. Llorente, Fernando López

**Affiliations:** 1 Department Otolaryngology, Hospital Universitario Central de Asturias, 33011 Oviedo, Spain; 2 Department Head and Neck Oncology, Instituto de Investigación Sanitaria del Principado de Asturias, 33011 Oviedo, Spain; 3 Department Pathology, Hospital Universitario Central de Asturias, 33011 Oviedo, Spain; 4 Department Translational Research and of New Technologies in Medicine and Surgery, University of Pisa, 56126 Pisa, Italy

**Keywords:** sinonasal cancer, poorly differentiated tumors, CD8+ TILs, PD-L1, MSI, immunotherapy

## Abstract

The sinonasal cavities harbor a wide variety of rare cancer types. Histopathological classification can be challenging, especially for poorly differentiated tumors. Despite advances in surgery and radio-chemotherapy, the 5-year survival rate is still very low. Thus, there is an unmet clinical need for new therapeutic options. We retrospectively evaluated poorly differentiated tumors of 9 different histological subtypes from 69 patients who had received conventional treatments for the presence of CD8+ tumor-infiltrating lymphocytes (TILs), as well as the expression of PD-L1 and microsatellite instability (MSI) markers MLH1, MSH2, MSH6 and PMS2, as biomarkers for immunotherapy. CD8+ TILs were present in 23/69 (33%) cases, PD-L1 expression was observed in 23/69 (33%), and markers for MSI positivity in 5/69 (7%) cases. CD8+ TILs correlated with PD-L1 positivity, while both were mutually exclusive with MSI markers. None of the biomarkers were associated with clinical features as age, gender or tumor stage. Cases with CD8+ TILs and PD-L1 positivity showed a tendency toward worse disease-specific survival. Immune checkpoint inhibitors are emerging as new options for treatment of many tumor types. Our results indicate that also a substantial subset of patients with poorly differentiated sinonasal tumors may be a candidate to be treated with this promising new therapy.

## 1. Introduction

Approximately 5% of all head and neck cancers arise in the sinonasal cavities [1]. They represent a wide histological diversity, each with their distinctive etiology, epidemiology, clinical and genetic characteristics [1,2]. About 70% are sinonasal squamous cell carcinoma (SNSCC) and intestinal-type adenocarcinoma (ITAC), and the remaining 30% are made up by a miscellany of poorly differentiated entities consisting of many epithelial cancer types, including the highly heterogeneous categories of undifferentiated carcinoma (SNUC), neuroendocrine carcinoma (SNEC) and high-grade non-ITAC (HG-non-ITAC) [3,4,5,6], but also non-epithelial tumors such as mucosal melanoma, several types of sarcoma, olfactory neuroblastoma and even hematological neoplasms. Together, they are termed poorly differentiated sinonasal tumors, sometimes also small round blue cell sinonasal tumors [3,4,5,6,7,8,9,10].

A growing number of these tumors can now be diagnosed by molecular genetic analysis, such as NUT carcinoma by chromosomal translocation t (15;19) NUT-BRD4, HPV-related multiphenotypic sinonasal carcinoma by high-risk HPV in the absence of the t (6;9) MYB-NFIB rearrangement, and subsets of SNSCC by EGFR exon 20 mutation and by DEK::AFF2 fusions [5,6,7,8]. New tumor entities previously classified as SNUC include SMARCB1-deficient carcinoma, SMARCA4-deficient carcinoma and possibly also IDH2 mutant SNUC [6,11,12].

A common factor of sinonasal tumors is their very low incidence, making it difficult for the clinician not only to reach a correct diagnosis, but also to develop and test treatment options. Despite advances in endoscopic surgery, precision radiotherapy, heavy ion radiotherapy, and induction chemotherapy, the 5-year survival rate remains less than 50%. For SNEC, SNUC, and NUT carcinoma prognosis is counted in months [13,14,15]. Local recurrence often occurs within two years of follow-up and is the main cause of mortality. Standard treatment is surgery combined with radiation, in SNUC additional chemotherapy may be beneficial [16,17,18].

To improve these outcomes, new treatment options for neoadjuvant, concomitant or adjuvant therapy are necessary, and immunotherapy may be such an option. Immune checkpoint inhibitors have improved outcomes for different types of solid tumors, including head and neck squamous cell carcinoma, non-small cell lung cancer and oesophageal squamous cell carcinoma [19,20,21]. Over recent years, several biomarkers as predictors of immunotherapeutic efficacy have been studied, the most important being CD8-positive tumor-infiltrating lymphocytes (CD8+ TILs), PD-L1 expression on tumor cells, tumor mutational burden (TMB) and microsatellite instability (MSI).

Previous studies on sinonasal tumors have indicated PD-L1 expression in 26% of ITAC and 30–46% PD-L1 expression in SNSCC [22,23,24,25,26]. A high presence of CD8+ TILs was reported in 8% of ITAC [21], 19–57% of SNSCC [24,25,26], while in olfactory neuroblastoma (ONB), they were much less frequent and predominantly found in the stromal compartment [27,28]. The majority of these studies on ITAC, SNSCC and ONB showed CD8+ TILs to be associated with better prognosis, while PD-L1 expression on tumor cells or macrophages generally did not carry prognostic value [22,24,26,28].

MSI is due to defects in the genes involved in the MMR (mismatch repair) system, such as MLH1, MSH2, MSH6 and PMS2. Although it was initially described in patients with sporadic colon cancer and hereditary non-polyposis colorectal cancer, it has now been demonstrated in many types of tumors. In two recent studies using MLH1, MSH2, MSH6 and PMS2 staining as surrogate markers of MSI, 3.2% (4/125 cases) and 2.3% (3/131 cases) were found to be MSI positive, indicated by loss of or reduced PMS2 and MLH1 [26,29]. In addition, analyzing 10 SNSCC cell lines by multiplex PCR of a reference panel with five nearly monomorphic mononucleotide markers BAT25, BAT26, D5S346, D2S123, D17S250, one cell line, SCCNC5, was found to be MMR-deficient. The same cell line was also determined to be TMB-high by whole exome sequencing [29]. Finally, Garciá-Martinez et al. reported MSI in 21% (5/24 cases) SNSCC and 2% (1/41 cases) ITAC [30].

To our knowledge, apart from SNSCC, ITAC and ONB, no other sinonasal tumor types have been studied with regard to biomarkers for immunotherapy. The aim of this study was to evaluate the presence of CD8+ TILs, PD-L1 expression and the expression of MLH1, MSH2, MSH6 and PMS2 as surrogate markers of MSI, in a series of poorly differentiated sinonasal carcinomas who had received conventional treatments, as possible indicators for treatment with immunotherapy. In addition, results were correlated with clinico-pathological and follow-up data.

## 2. Materials and Methods

### 2.1. Patients and Samples

Samples from poorly differentiated sinonasal carcinomas of 69 patients who had received conventional treatments were collected from the Hospital Universitario Central de Asturias (HUCA), Oviedo, Spain; from the VU Medical Center (VUmc), Amsterdam, the Netherlands, and from University Medical Center Utrecht (UMC), Utrecht, the Netherlands. All experimental protocols were approved by the Institutional Ethics Committee of the Hospital Universitario Central de Asturias and by the Regional CEIC from Principado de Asturias (approval numbers 07/16 for project CICPF16008-HERM, 83/17 for project PI17/00763 and 2020.048 for project PI19/00191). All methods were carried out in accordance with the guidelines of the Institutional Ethics Committee of the Hospital Universitario Central de Asturias. Informed consent was obtained from all patients.

**Figure 1:**
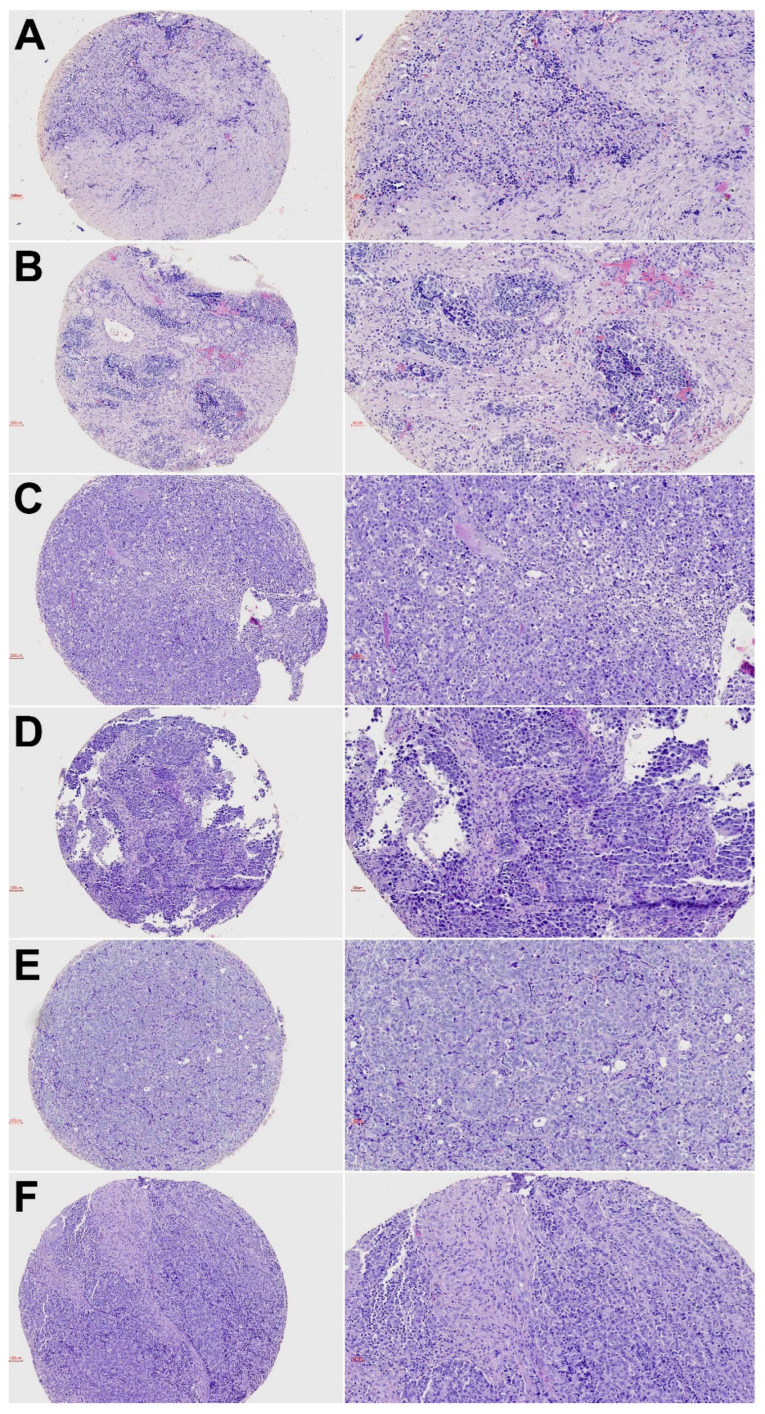
Hematoxylin and eosin stainings of representative cases from tumor subtypes ONB (**A**), SNEC (**B**), SNUC (**C**), PD-SNSCC (**D**), HG-non-ITAC (**E**) and solid-type ITAC (**F**). Left column ×100 and right column ×200 magnification.

Our cohort included 14 olfactory neuroblastoma (ONB), 6 neuroendocrine carcinoma (SNEC), 25 (SNUC), 6 poorly differentiated squamous cell carcinoma (PD-SNSCC), 5 HG-non-ITAC, 10 solid-type ITAC, 1 SMARCB1-deficient carcinoma, 1 SMARCA4-deficient carcinoma and 1 NUT carcinoma. Representative photomicrographs of hematoxylin and eosin staining of the tumor types are given in Figure 1 and Supplementary Figure S1. Of the 65 (54%) patients, 35 were male and 30 (46%) female; the mean age was 56 years, ranging from 20 to 83 among 63 patients. The distribution of disease stage according to the TNM system for tumor classification [25] was known for 64 patients: 8 (12%) were stage I, 24 (38%) stage II, 8 (12%) stage III, 20 (31%) stage IVa and 4 (6%) were stage IVb. The clinical characteristics according to specific tumor type are presented in Table 1.

The initial diagnoses of all cases were revised by an experienced pathologist (AF). All the available slides were reviewed in each case, and tumors were diagnosed using the diagnostic criteria described in the fourth edition of the World Health Organization Classification [1]. Additional immunohistochemical stainings CK5/6, CK20, CDX2, p40, p16, synaptophysin, chromogranin, NUT, SMARCB1, and SMARCA4 were applied when necessary. As none of the tumors stained positive for p16, no further testing for human HPV was performed. In addition, IDH2 mutation was observed in 1/6 (17%) SNEC, 9/25 (36%) SNUC, 1/6 (17%) PDSNSCC and 2/5 HG-non-ITAC. These analyses were previously published by [31].

### 2.2. Immunohistochemical Analysis

Tumor samples which were obtained from biopsy or surgery were fixed with formalin and embedded in paraffin. When several paraffin blocks were available for the same tumor, the most representative block was selected. Tissue microarray blocks were prepared using the Beecher Tissue Microarrayer (Beecher Instruments, Silver Spring, MD, USA). In total, 4 blocks were constructed, containing three 1 mm cores from different areas of 69 tumors [32].

Immunohistochemistry was performed on an automatic staining workstation (Dako Autostainer Plus; DakoCytomation, Glostrup, Denmark) with antigen retrieval by EnVision FLEX + Mouse (DakoCytomation, Glostrup, Denmark) over 20 minutes. The following antibodies were applied: anti-CD8 clone C8/144B, IR623 (Prediluted monoclonal mouse, DAKO, Glostrup, Denmark), anti-PD-L1 clone E1L3N (1/100 monoclonal rabbit, Cell Signalling Technology, Cambridge, UK), and MSI markers anti-MLH1 clone M1, anti-PMS2 clone A16-4, anti-MSH2 clone G219–1129 and anti-MSH6 clone SP93 (Ventana Roche, Tucson, AZ, USA). The slides were evaluated in a double-blind manner by three observers (EVF, BV and MAH), and discrepancies between the observers were resolved by a consensus review after simultaneous reevaluation.

CD8+ TIL scoring was adapted from Fuchs et al. [33], who defined low, intermediate and high CD8+ TILs as 1–10%, 10–50% and >50%, adding another group with 0%. However, in two of our previous studies [22,25] we found that survival was similar in the two groups 10–50%/>50% and 0%/1–10%; therefore, we simplified the present scoring to low (<10%) or high (>10%) of the cells present in the stromal or in the intratumoral compartment. Staining for PD-L1 was considered positive when >5% of the tumor cells showed membranous and/or cytoplasmic staining, in accordance with most studies in the field, including clinical immunotherapy trials [34,35]. Regarding MSI, tumors that showed significantly reduced nuclear staining compared to intratumoral immune cells or tumors that displayed complete loss of an MMR protein were classified as MSI.

### 2.3. Statistical Analysis

Pearson’s chi-squared test and Fisher’s exact test were used to test possible associations between CD8+ TILs and PD-L1 expression and MSI presence and various clinicopathological factors. Univariate Kaplan–Meier curves were plotted to assess the relations of CD8+ TILs, PDL-1 expression and MSI to overall, disease-specific and disease-free survival using the log-rank-test. *p*-values < 0.05 were considered to indicate statistically significant. Statistical analysis was carried out with the use of SPSS Base, version 15.0 and SPSS Advanced models, version 15.0 (SPSS Inc., Chicago, IL, USA) software. 

## 3. Results

### 3.1. Clinical Features and Follow-Up

**Figure 2:**
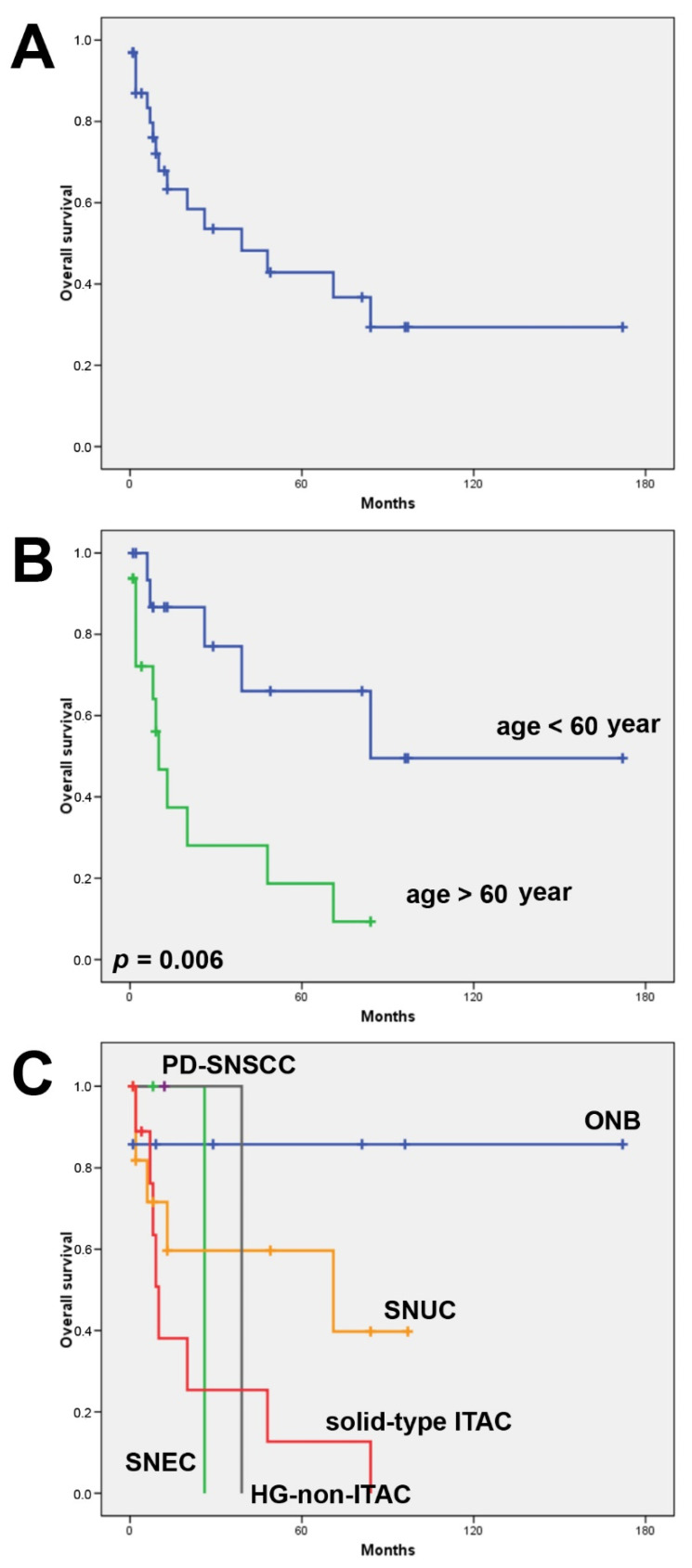
Kaplan–Meier overall survival analysis. All cases (**A**), according to age (**B**) and according to tumor subtype (**C**).

All 69 patients were treated by surgery and 15 patients also received radiotherapy. The mean follow-up available of 33 patients was 31 months (range 1–172). During this period, 22 (67%) patients developed a recurrence or metastasis. At the time of writing, 17 (52%) patients remained alive, 13 (39%) died of disease and 3 (9%) died of other causes. The 5-year overall survival was 43% and the 5-year disease-free survival was 34%. Tumor stage was not correlated with overall or disease-free survival. Patients of 60 years of age and older showed worse overall survival (Log rank 7.661, *p* = 0.006), while there was no age difference regarding disease-free survival (Figure 2). Comparing the histological subtypes, ONB and SNUC showed the most favorable clinical outcomes with a 5-year overall survival of 86% and 60%, respectively. None of the patients with SNEC, HG-non-ITAC and solid-type ITAC reached 5-year overall survival (Table 1). Unfortunately, for PD-SNSCC, SMARCB1-deficient carcinoma, SMARCA4-deficient carcinoma and NUT carcinoma, no follow-up data were available.

### 3.2. CD8+ Tumor Infiltrating Lymphocytes

**Figure 3:**
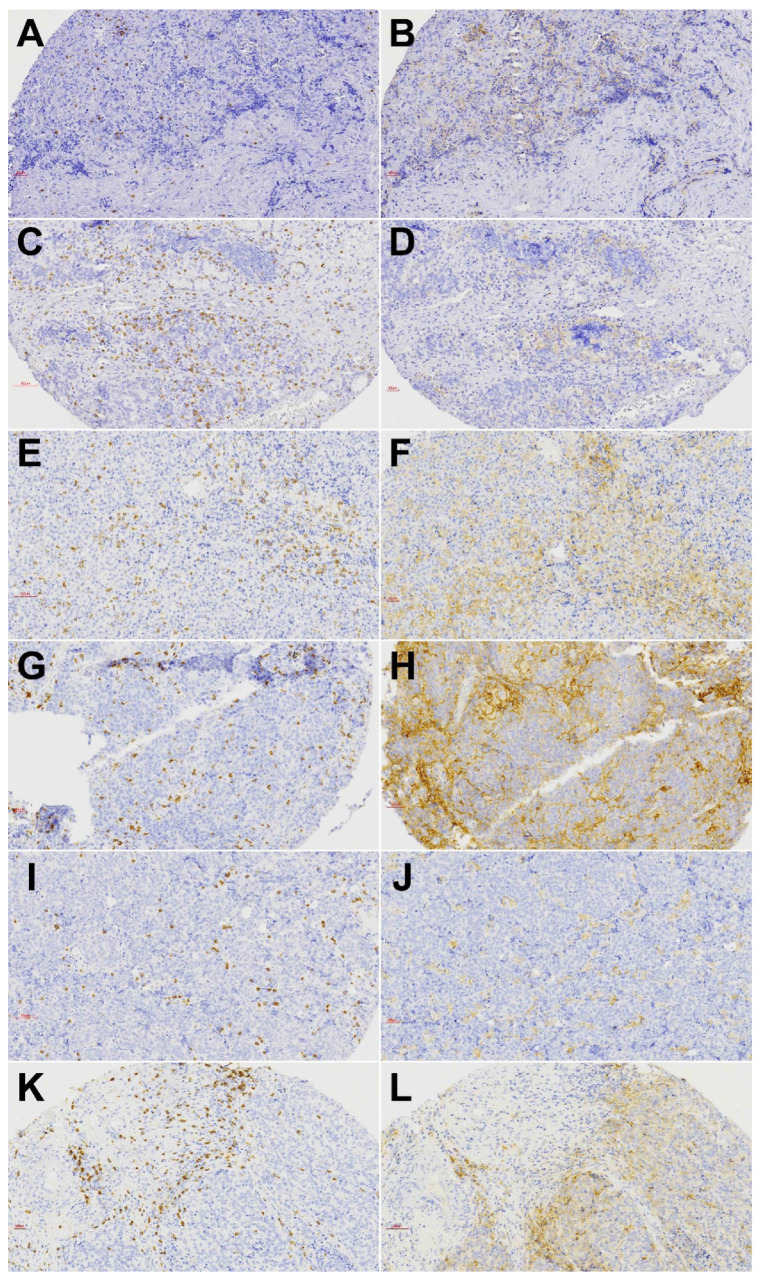
Immunohistochemical staining of CD8+ TILs and PD-L1-expressing tumor cells of tumor subtypes ONB (**A**,**B**), SNEC (**C**,**D**), SNUC (**E**,**F**), PD-SNSCC (**G**,**H**) HG-non-ITAC (**I**,**J**) and solid-type ITAC (**K**,**L**). Left column: CD8+ TILs and right column: PD-L1 expression. All images ×200 magnification.

**Figure 4:**
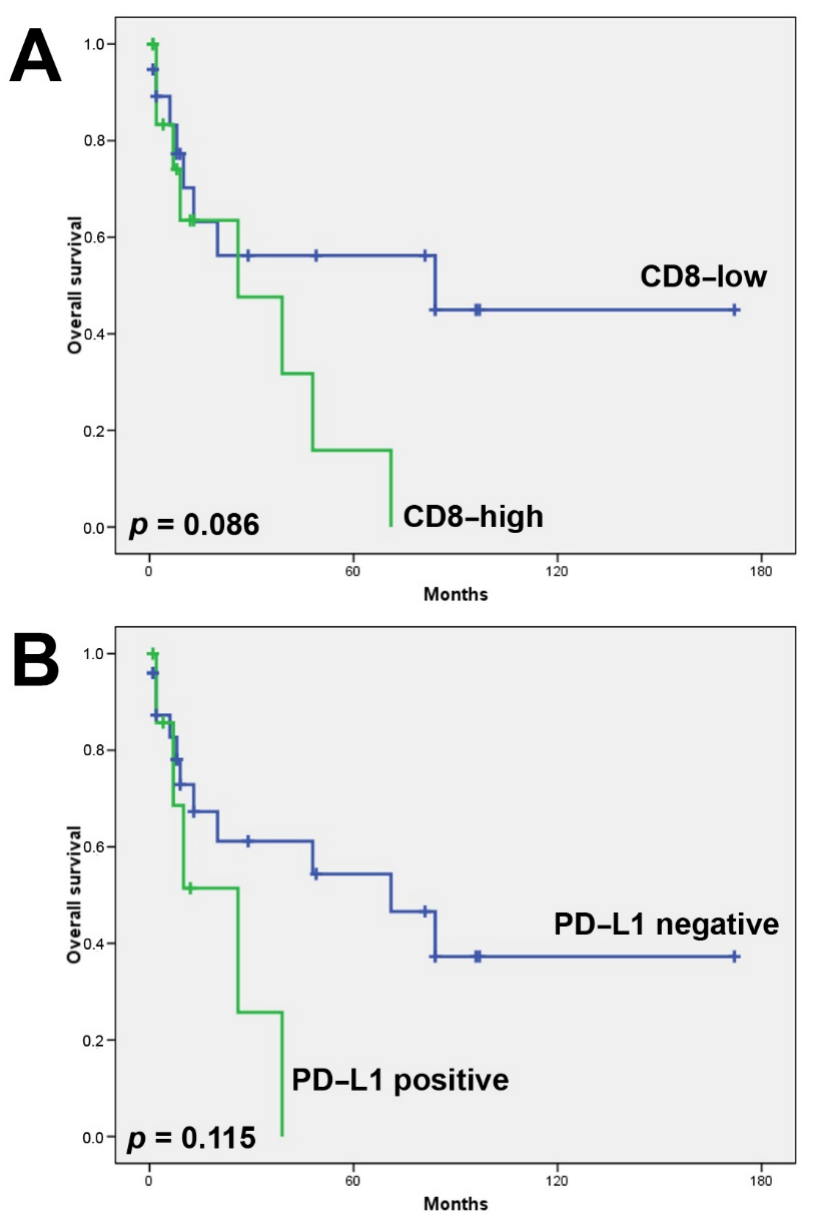
Kaplan–Meier overall survival analysis according to CD8+ TIls (**A**) and according to PD-L1 expression (**B**).

A total of 23 of 69 (33%) tumors showed a high presence of CD8+ TILs, either in the tumor or stromal compartment. In 21/23 cases, CD8+ TILs were present in both compartments. CD8+ TILs were most frequent in solid-type ITAC and HG-non-ITAC (60% of tumors), followed by SNUC (34%), PDSCC (33%), ONB (21%) and SNEC (17%). SMARCB1-deficient carcinoma, SMARCA4-deficient carcinoma and NUT carcinoma were devoid of CD8+ TILs (Table 2). Examples of CD8 stainings are given in Figure 3 and Supplementary Figure S1. A total of 30% of IDH2-mutated versus 13% IDH2-wildtype tumors harbored CD8+ TILs; however, this difference was not significant (Fisher’s Chi2 *p* = 0.082). The presence of CD8+ TILs was not related to age, tumor stage or clinical outcome, although there was a tendency toward worse overall survival (Figure 4).

### 3.3. PD-L1 Expression

A total of 23 of 69 (33%) tumors showed membranous PD-L1 staining in >5% of tumor cells. The staining pattern was diffuse in the majority of cases, whereas HG-non-ITAC mostly demonstrated focal expression (Figure 3). PD-L1 expression was most frequently observed in PDSNSCC (83%), HG-non-ITAC (80%) and solid-type ITAC (60%). In SNEC, SNUC and ONB this was 33%, 16% and 14%, respectively (Table 2). The one case of SMARCB1-deficient carcinoma was also PD-L1 positive, while SMARCA4-deficient carcinoma and NUT carcinoma showed no expression (Supplementary Figure S1). PD-L1 positivity in IDH2-mutated was seen in 26% and in IDH2-wildtype tumors in 15% of cases. A total of 13 of 23 (56%) PD-L1 positive versus 10/46 (22%) PD-L1 negative tumors also carried CD8+ TILs (Fisher’s Chi2 *p* = 0.006). We found no correlation between PD-L1 expression and age or tumor stage. PD-L1 positive cases did demonstrate both worse overall (Figure 4) and disease-free survival (not shown); however, this did not reach significance.

### 3.4. Microsatellite Instability

**Figure 5:**
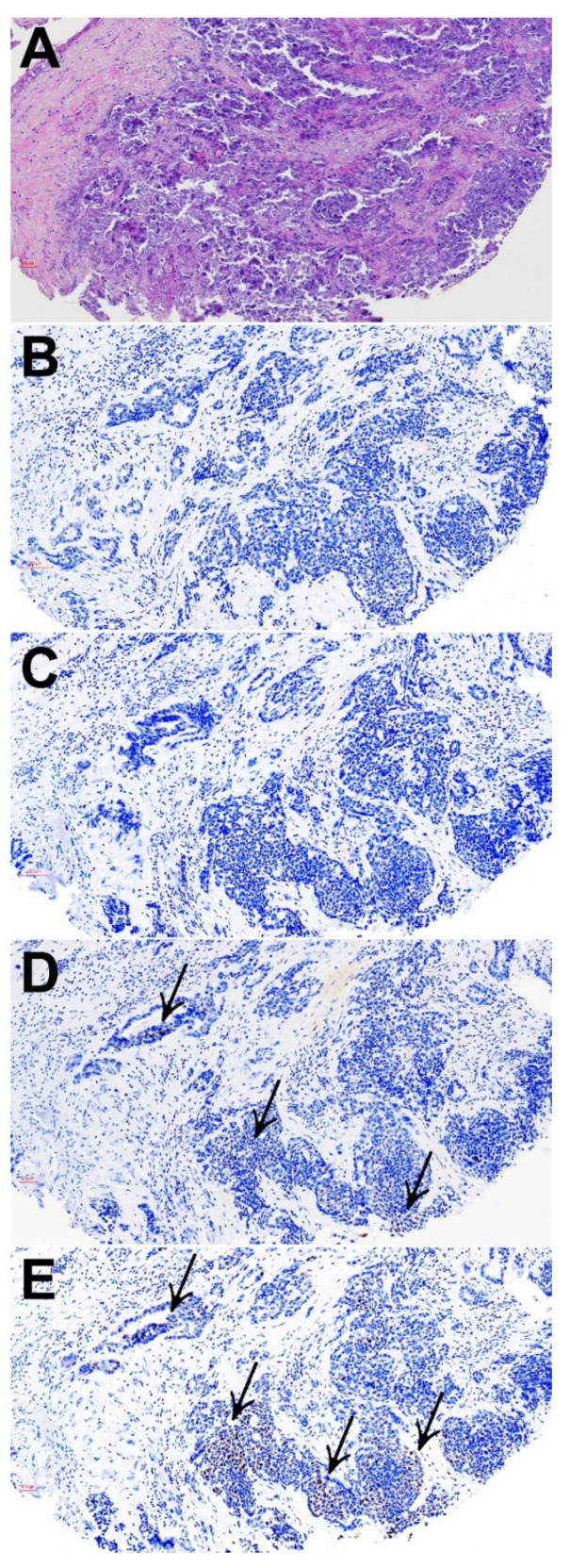
Representative MSI-positive SNUC showing stainings of hematoxylin and eosin (**A**), PMS2 (**B**), MLH1 (**C**), MSH2 (**D**) and MSH6 (**E**). Arrows indicate small patches of positive cells. All images ×200 magnification.

We applied PMS2, MLH1, MSH2, and MSH6 staining as surrogate markers of MSI and found 5/69 (7%) cases positive, two were SNEC, two SNUC and one was HG-non-ITAC. Four tumors demonstrated complete absence and one reduced expression of PMS2, accompanied by complete loss of MLH1 in two cases and reduced MLH1 in two cases, while in one case PMS2 was the only lost of the four MSI markers. One case showed complete absence of all four markers, and in one case shown in Figure 5, PMS2 and MLH1 were lost and MSH2 and MSH6 reduced (Supplementary Table S1). All five MSI positive cases were IDH2-wildtype and did not show PD-L1 expression. One MSI positive case (SNUC) did harbor CD8+ TILs. MSI positivity was not related to age or stage; correlation with survival was not analyzed, as follow-up information was only available of two MSI-positive cases.

## 4. Discussion

Standard treatment of poorly differentiated sinonasal carcinomas consists of surgery and radiotherapy, in some cases complemented with histo-type driven chemotherapy [15,17]. In spite of advances in these approaches, these tumors still carry a very poor prognosis. Moreover, being rare cancers, there are few studies on alternative therapeutic options. CD8+ TILs, PD-L1 expression and MSI are recognized biomarkers for treatment with immune checkpoint inhibitors and have been studied in various subtypes of sinonasal cancer [22,23,24,25,26,27,28,29,30]. Other biomarkers include TMB and gene expression profiling of immune pathways and the first studies on sinonasal cancer indeed indicated their potential to identify patients with SNSCC, SNUC or high-grade carcinomas that may benefit from immunotherapy [29,36,37]. In this study, we focused on CD8+ TILs, PD-L1 expression and MSI.

Considering the whole cohort of 69 tumors, we found 33% to have CD8+ TILs, 33% with PD-L1 expression and 7% as MSI-positive. In nearly all cases, CD8+ TILs were present both in the tumor and the stromal compartment and correlated with PD-L1 positivity, which is in agreement with many previous studies. In contrast with previous studies on MSI-positive tumors, we found MSI positivity to be mutually exclusive with CD8+ TILs and nearly also with PD-L1 expression [38,39,40]. This may be due to the fact that we only applied PMS2, MLH1, MSH2, and MSH6 immunohistochemistry without the possibility to confirm our results by multiplex PCR. Additionally, the low number of 5 MSI-positive cases and the whole cohort of 69 is a limitation to our observation.

Comparing the different tumor types, CD8+ TILs were observed most frequently (60% of cases) in the adenocarcinoma subtypes HG-non-ITAC and solid-type ITAC, which is higher than previous studies on sinonasal tumors reporting 19–50% in SNSCC [24,25,26,41,42] and 19% in ITAC [22]. In CD8+ TILs were much less frequent and were predominantly found in the stromal compartment [27,28]. Additionally, PD-L1 expression was the most frequent (50–83% of cases) in HG-non-ITAC and solid-type ITAC, together with PDSNSCC. Again, these percentages are higher than the 30–46% observed in SNSCC [24,25,26], the 26% in ITAC [22] and the 20% in ONB [28]. Our finding of 7% of cases with MSI are in agreement with earlier immunohistochemical and multiplex PCR studies on sinonasal tumors, reporting a 2–3% in SNSCC and no MSI in other tumor subtypes. One study using multiplex PCR demonstrated MSI in 21% of ITAC, but there are no studies confirming this finding [30].

Although follow-up information in our study was very limited and the statistical power of the survival analysis low, our results indicated a tendency toward worse clinical outcome for tumors with CD8+ TILs. This finding contrast with a number of studies on ITAC, ONB and SNSCC have indicated CD8+ TILS as indicators of better survival [22,24,26,27,42,43]. Indeed, a positive relation between CD8+ TILs and favorable prognosis is found in the majority of cancer types [44].

However, there are also reports that associated CD8+ TILs with worse survival in SNSCC [25], and in non-small cell lung cancer [45,46]. Discrepancies may be due to the use of different cutoff points and differences in immunostaining protocols and also to differences in patient cohorts and treatment schemes.

Our data also suggest a possible association between PD-L1 expression and worse clinical outcome. This is in accordance with a similar study on SNSCC by Hongo et al. [26], although other studies on sinonasal tumors including ITAC, ONB and SNSCC reported no prognostic value for PD-L1 [22,23,24,28]. Additionally, in other solid tumor types, such as head and neck squamous cell carcinoma and lung cancer, contradictory data on the prognostic role of PD-L1 expression have been presented, with either relation to poor prognosis, to better survival or no relation at all [47,48,49,50,51]. Tumor cells express PD-L1 to escape the immune system. We found that the presence of CD8+ TILs correlated with tumoral PD-L1 expression; therefore, the tendency toward worse clinical outcome for tumors with PD-L1 expression and CD8+ TILs in our series may indicate that these CD8+ TILs are dysfunctional, exhausted cells.

We found 5/69 tumors with an indication for MSI positivity, four with complete loss of PMS2 together with lost/reduced MLH1 expression. In general, MSI positivity is considered a predictor of favorable clinical outcome. With follow-up information of only two of the MSI-positive cases in our cohort, no meaningful assessment of prognostic vale was possible; however, these patients had a very poor survival of 2 and 6 months. Our cohort included one case each of SMARCB1-deficient carcinoma, SMARCA4-deficient carcinoma and NUT carcinoma, unfortunately without follow-up information. 

The FDA has approved MSI status, analyzed by immunohistochemistry or by multiplex PCR, as predictor for the efficacy of immunotherapy of unresectable or metastatic cancer, irrespective of the primary site of origin [52]. More specifically, for head and neck squamous cell carcinoma, the PD-1 inhibitor pembrolizumab is approved as a single agent for patients whose tumors express PD-L1, and nivolumab and pembrolizumab are also approved for second-line treatment of recurrent and metastatic tumors [19]. The degree of infiltration of CD8+ T cells is correlated with improved response rates to anti-PD-1/PD-L1 agents in HNSCC [53]. There are few clinical studies on rare head and neck cancer subtypes, such as the sinonasal tumors presented in our study. Park et al. reported favorable response to PD-1 inhibitors in 11 recurrent/metastatic SNSCC, even more so than studies on non-SNSCC head and neck squamous cell carcinoma have shown, although a direct comparison was not possible. Notably, the response was not related to PD-L1 expression [54]. Another clinical study with nivolumab (anti-PD-1) included 18 sinonasal tumors of different histological subtypes and showed response rates comparable to those reported for head and neck squamous cell carcinoma, with the highest efficacy against high-grade non-squamous cell sinonasal tumors [55]. Additionally, a clinical study with nasopharyngeal carcinoma patients has shown the promising efficacy of atezolizumab (anti-PD-L1) [56].

Our results on poorly differentiated sinonasal carcinomas revealed that at least one of the immunotherapeutic biomarkers >10% CD8+ TILs, >5% PD-L1 expression or MSI-positivity occurred in 39% (27/69) of cases. For immunotherapy to be most effective, tumors ideally should express PD-L1 and have tumor-activated CD8+ TILs [57], and this is what we observed in 19% (13/69) cases. MSI positivity in 5 cases was mutually exclusive with these 13 cases, which would bring the total number eligible for immunotherapy to 18 patients, 26% of the cohort. 

The three cases SMARCB1-deficient carcinoma, SMARCA4-deficient carcinoma and NUT carcinoma may be given special attention. Recent studies suggest that SMARCB1 and SMARCA4 deficiencies lead to enhanced levels of TILs and PD-L1, despite low TMB [58,59,60]. Our two cases showed the absence of CD8+ TILs, whereas the SMARCB1-deficient carcinoma did express PD-L1. Nevertheless, a clinical study on metastastic pancreatic SWI/SNF cancer showed good responses to immunotherapy in 8/9 patients, independent of MSI or TMB status or PD-L1 expression [61]. Finally, preclinical studies on mice models of pulmonary NUT carcinoma indicated the synergistic effects of immunotherapy combined with BET-inhibitor [62]. These early promising results may be relevant for sinonasal SWI/SNF and NUT carcinomas as well.

Our study has several limitations. First, the series of cases is not homogeneous. Although all tumors are considered poorly differentiated, they do belong to distinct sinonasal cancer subtypes. Second, due to the fact that these are rare tumors, we could only analyze a relatively low number of cases, and thus more studies are necessary. Finally, the patients in our retrospective cohort had received conventional treatments that did not include immunotherapy, so it was not possible to correlate response to therapy with CD8+ TILs and PD-L1 expression status or to find an explanation as to why cases with CD8+ TILs had worse clinical outcomes. However, this negative correlation does not preclude that these patients would be less responsive to immune checkpoint inhibitors.

To conclude, immune checkpoint inhibitors represent new options for treatment for an increasing number of tumor types. Our results on CD8+ TILS, PD-L1 expression and MSI indicate that also a substantial subset of patients with poorly differentiated sinonasal tumors may be candidates to be treated with this promising new therapy.

## Figures and Tables

**Table 1:** Clinical features of all tumors.

Clinical Characteristics	All	ONB	SNEC	SNUC	PD-SNSCC	HG-Non-ITAC	Solid-Type ITAC	SmarcB1-Def Carcinoma	SmarcA4-Def Carcinoma	NUT Carcinoma
All	69	14	6	25	6	5	10	1	1	1
Age *	56 (20–83)	47 (20–69)	60 (49–77)	56 (34–83)	67 (57–77)	37 (29–47)	67 (49–82)	59	NA	40
Sex										
Male	35/65 (54)	3/13 (23)	2/6 (33)	13/27 (52)	4/5 (80)	3/4 (75)	9/10 (90)	1 (100)	NA	0 (0)
Female	30/65 (46)	10/13 (77)	4/6 (67)	12/27 (48)	1/5 (20)	1/4 (25)	1/10 (10)	0 (0)	NA	1 (100)
Disease stage										
I	8/64 (12)	0/13 (0)	0/6 (0)	0/27 (0)	4/5 (80)	2/3 (67)	2/10 (20)	0 (0)	NA	0 (0)
II	24/64 (38)	7/13 (54)	4/6 (67)	10/27 (40)	1/5 (20)	1/3 (33)	0/10 (0)	0 (0)	NA	1 (100)
III	8/64 (12)	2/13 (15)	1/6 (17)	2/27 (8)	0/5 (0)	0/3 (0)	3/10 (30)	0 (0)	NA	0 (0)
IVa	20/64 (31)	4/13 (31)	0/6 (0)	13/27 (52)	0/5 (0)	0/3 (0)	2/10 (20)	1 (100)	NA	0 (0)
IVb	4/64 (6)	0/13 (0)	1/6 (17)	0/27 (0)	0/5 (0)	0/3 (0)	3/10 (30)	0 (0)	NA	0 (0)
Recurrence	19/32 (59)	5/7 (71)	2/2 (100)	4/12 (33)	NA	1/1 (100)	7/10 (70)	NA	NA	NA
Follow up *	31 (1–172)	56 (1–172)	17 (8–26)	29 (1–97)	12 (12–12)	39 (39–39)	19 (1–84)	NA	NA	NA
Patient status										
Alive	17/33 (52)	6/7 (86)	1/2 (50)	7/12 (58)	1/1 (100)	0/1 (0)	2/10 (20)	NA	NA	NA
DOD	13/33 (39)	1/7 (14)	1/2 (50)	3/12 (25)	0/1 (0)	1/1 (100)	7/10 (70)	NA	NA	NA
DOC	3/33 (9)	0/7 (0)	0/2 (0)	2/12 (17)	0/1 (0)	0/1 (0)	1/10 (10)	NA	NA	NA

Legend. *: mean (range); NA: not available; DOD: died of disease; DOC: died of other causes.

**Table 2:** CD8+ TILs, PD-L1 and MSI scores according to tumor subtype.

	CD8+ TILs > 10%	PD-L1 > 5%	MSI-Positive
ONB	3/14 (21%)	2/14 (14%)	0/14 (0%)
SNEC	1/6 (17%)	2/6 (33%)	2/6 (33%)
SNUC	8/25 (32%)	4/25 (16%)	2/25 (8%)
PD-SNSCC	2/6 (33%)	5/6 (83%)	0/6 (0%)
HG-non-ITAC	3/5 (60%)	4/5 (80%)	1/5 (20%)
Solid-type ITAC	6/10 (60%)	5/10 (50%)	0/10 (0%)
SmarcB1-def carcinoma	0/1 (0%)	1/1 (100%)	0/1 (0%)
SmarcA4-def carcinoma	0/1 (0%)	0/1 (0%)	0/1 (0%)
NUT carcinoma	0/1 (0%)	0/1 (0%)	0/1 (0%)
All	23/69 (33%)	23/69 (33%)	5/69 (7%)

## Data Availability

Data are available with the corresponding upon request.

## Supplementary Materials

The following supporting information can be downloaded at: https://www.mdpi.com/article/10.3390/biomedicines10092205/s1, Table S1. PMS2, MLH1, MSH2 and MSH6 staining pattern in 5 cases designated as MSI positive. Figure S1. Representative images of SMARCB1-deficient carcinoma (A–D), SMARCA4-deficient carcinoma (E–H) and NUT carcinoma (I–L) showing hematoxylin and eosin staining (A,E,I), SmarcB1 expression (B), SmarcA4 expression (F) and NUT1 expression (J), as well as CD8+ TILs (C,G,K) and PD-L1 (D,H,L).
