# Supplementary material for: Biomarkers for Immunotherapy in Poorly Differentiated Sinonasal Tumors
Source: Biomedicines. 2022 Sep 6;10(9):2205. doi: 10.3390/biomedicines10092205 (PMC9496628; doi:10.3390/biomedicines10092205)

SUPPLEMENTARY MATERIALS

Biomarkers of response to immunotherapy in poorly differentiated sinonasal carcinomas

Eva Villanueva-Fernández1, Mario A. Hermsen2, Laura Suárez-Fernández2, Blanca Vivanco3, Alessandro Franchi4, Rocío García-Marín2, Virginia N. Cabal2, Helena Codina-Martínez2, Sara Lucila Lorenzo-Guerra2, José L. Llorente1, Fernando López1.

Table S1. PMS2, MLH1, MSH2 and MSH6 staining pattern in 5 cases designated as MSI positive.

	PMS2	MLH1	MSH2	MSH6
**SNEC patient 1**	negative	reduced	positive	positive
**SNEC patient 2**	negative	negative	negative	negative
**SNUC patient 1**	negative	reduced	reduced	reduced
**SNUC patient 2**	negative	negative	reduced	reduced
**HG-non-ITAC**	reduced	reduced	positive	positive

Figure S1. Representative images of SMARCB1-deficient carcinoma (**A**–**D**), SMARCA4-deficient carcinoma (**E**–**H**) and NUT carcinoma (**I**–**L**) showing hematoxylin and eosin staining (**A**,**E**,**I**), SmarcB1 expression (**B**), SmarcA4 expression (**F**) and NUT1 expression (**J**), as well as CD8+ TILs (**C**,**G**,**K**) and PD-L1 (**D**,**H**,**L**). All images ×200 magnification.

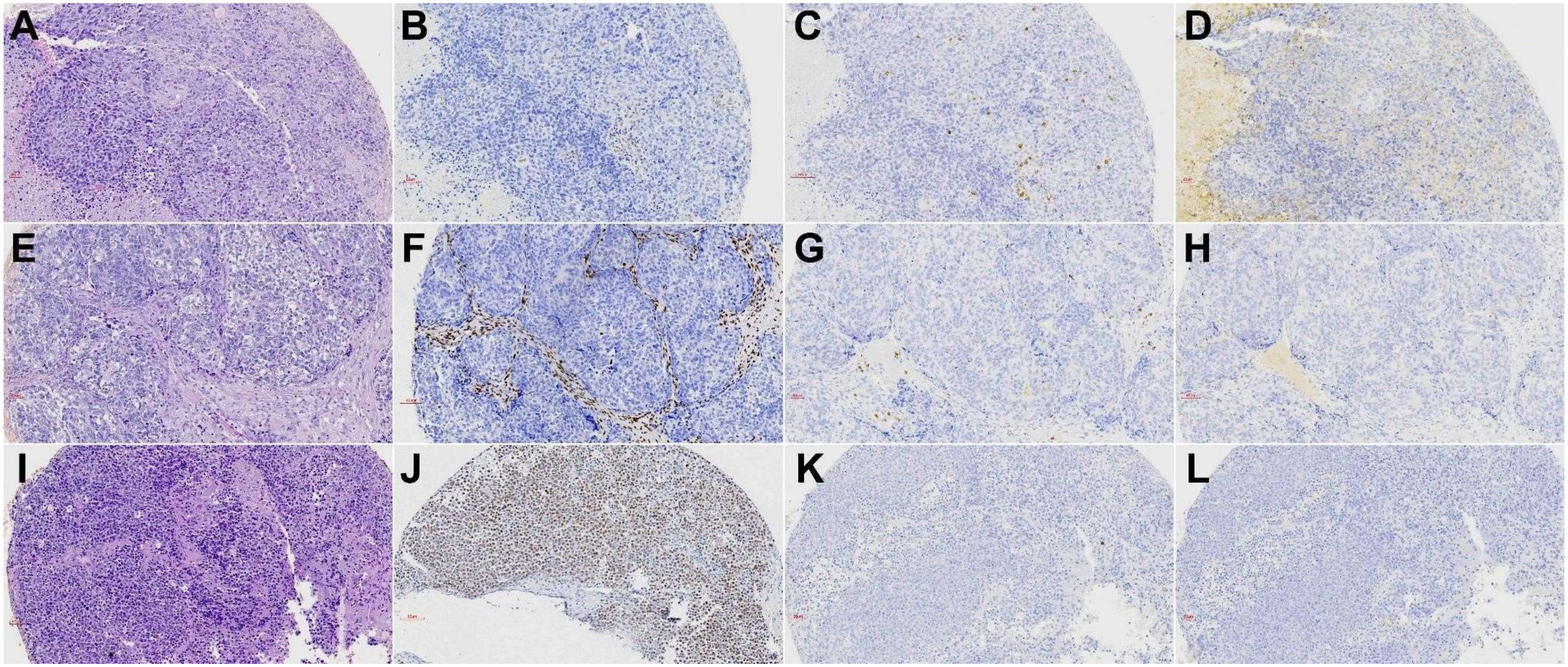

Supplement: Supplementary file 1 [file biomedicines-10-02205-s001.zip › biomedicines-1859832-supplementary.pdf]
